# Entropy Measures for Data Analysis: Theory, Algorithms and Applications

**DOI:** 10.3390/e21100935

**Published:** 2019-09-25

**Authors:** Karsten Keller

**Affiliations:** Institut für Mathematik, Universität zu Lübeck, D-23562 Lübeck, Germany; keller@math.uni-luebeck.de

**Keywords:** data analysis, entropy, entropy measures, automatic learning

Entropies and entropy-like quantities are playing an increasing role in modern non-linear data analysis and beyond. Fields that benefit from their application reach from diagnostics in physiology, for instance, electroencephalography (EEG), magnetoencephalography (MEG) and electrocardiography (ECG), to econophysics and engineering. During the last few years, classical concepts as approximate entropy and sample entropy have been supplemented by new entropy measures, like permutation entropy and its variants. Recent developments are focused on multidimensional generalizations of the concepts with a special emphasis on the quantification of coupling and the similarity between time series and system components behind them. One of the main future challenges in the field include finding a better understanding of the nature of the various entropy measures and their relationships, with the aim of adequate application including good parameter choices. The utilization of entropy measures as features in automatic learning and their application to large and complex data for such tasks as classification, discrimination and finding structural changes requires fast and well-founded algorithms. This issue is facing a different aspect of the use of entropy measures for data analysis in a wide sense, including those described.

Papers 1–3 discuss the problem of parameter choice mentioned and aspects related to it. Ahmadi et al. [1] investigate the sensitivity of sample entropy with respect to different parameters like, for example, tolerance size and sampling rate for gait data. Cuesta-Frau et al. [2] study parameter choice for permutation entropy, particularly embedding dimension and time series length, in the context of a lot of synthetic and real data sets. Here special emphasis is put on practical aspects of data analysis. In particular, the authors point out that in many cases permutation entropy can be used for shorter data sets than reported by other authors. In a certain sense complementary to the paper of Cuesta-Frau et al. [2], Piek and Keller [3] investigate parameter choice for permutation entropy and, more generally, ordinal pattern-based entropies from a computational and theoretical viewpoint. Fast algorithms are presented, possibilities and limits of the estimation of the Kolmogorov–Sinai entropy are discussed. A further aspect of the paper is the generation of artificial data for testing ordinal pattern methods.

The main objective of papers 4 and 5 is entropy-based feature extraction. Lu et al. [4] utilize approximate entropy, sample entropy, composite multiscale entropy and fuzzy entropy for identifying auditory object-specific attention from single-trial EEG signals by support vector machine (SVM)-based learning. For circuit fault diagnosis, He et al. [5] propose a new feature extraction method, which is mainly based on a measure called joint cross-wavelet singular entropy and a special dimension reduction technique. The obtained features are entered into a support vector machine classifier in order to locate faults. Besides feature extraction, direct applications of entropy for automatic learning is also addressed in this issue. Bukovsky et al. [6] discuss and further develop the recently introduced concept of learning entropy (LE) as a learning-based information measure, which is targeted at real-time novelty detection based on unusual learning efforts. For assessing the quality of data transformations in machine learning, Valverde-Albacete et al. [7] introduce an information-theoretic tool. They analyze performance of the tool for different types of data transformation, among them principal component analysis and independent component analysis. 

Papers 8–10 are devoted to the aspect of coupling and similarity analysis. For studying the Chinese stock market around the 2015 crash, Wang and Hui [8] utilize effective transfer entropy (ETE), which is an adaption of transfer entropy to limited and noisy data. From this base, they discuss and compare dependencies of 10 Chinese stock sectors during four characteristic time periods near the crash. In [9], Craciunescu et al. introduce a new measure for describing coupling in interconnected dynamical systems and test it for different system interactions. Besides such in model systems, real-life system interactions like between the El Niño Southern Oscillation, the Indian Ocean Dipole, and influenza pandemic occurrence are considered. Here, coupling strength is quantified by entropies of adjacency matrices associated to networks constructed. Wang et al. [10] use entropy-based similarity and synchronization indices for relating postural stability and lower-limb muscle activity. Their study is based on two types of signals, one measuring the centre of pressure (COP) in dependence on time and one being an electromyogram (EMG). The authors show high correlation of COP and the low frequency EMG and that the cheaper COP contains much information on the EMG.

The other four papers touch further interesting aspects of entropy measure use. Selvachandran et al. [11] consider complex vague soft sets (CCVS), defined as a hybrid model of vague soft sets and complex fuzzy sets, which is, for example, useful for the description of images. Some distance and entropy measures for CCVSs are axiomatically defined and relations between them are investigated. The work [12] of Pan et al. focuses on Dempster–Shafer evidence theory, which can be considered as a generalization of probability theory. A new belief entropy, measuring uncertainty in this framework, and its performance are discussed on the base of numerical experiments. García-Gutiérrez et al. [13] introduce a new model for the particle size distribution (PSD) of granular media, which relates two models known for a long time. For this purpose, a differential equation involving the information entropy is used. The interesting point is that experimental data can be considered as an initial condition for simulating a PSD. Last but not least, Liu et al. [14] demonstrate that entropy methods also can be helpful in solving nonlinear and multimodal optimization problems. They develop an algorithm based on the firefly algorithm and the cross-entropy method and report its good performance, especially powerful global search capacity precision and robustness for numerical optimization problems.
